# Real-world impact of bridging therapy on outcomes of ide-cel for myeloma in the U.S. Myeloma Immunotherapy Consortium

**DOI:** 10.1038/s41408-024-00993-0

**Published:** 2024-04-12

**Authors:** Aimaz Afrough, Hamza Hashmi, Doris K. Hansen, Surbhi Sidana, Chul Ahn, Lauren C. Peres, Danai Dima, Ciara L. Freeman, Omar Castaneda Puglianini, Mehmet H. Kocoglu, Shebli Atrash, Peter M. Voorhees, Leyla Shune, Joseph P. McGuirk, Gary Simmons, Douglas W. Sborov, James A. Davis, Gurbakhash Kaur, Aishwarya Sannareddy, Christopher J. Ferreri, Mahmoud R. Gaballa, Scott Goldsmith, Omar Nadeem, Shonali Midha, Charlotte B. Wagner, Frederick L. Locke, Krina K. Patel, Jack Khouri, Larry D. Anderson, Yi Lin

**Affiliations:** 1 Myeloma, Waldenstrom’s, and Amyloidosis Program, Hematologic Malignancies and Cellular Therapy Program, Simmons Comprehensive Cancer Center, UT Southwestern Medical Center, Dallas, TX USA; 2 Medical University of South Carolina, Charleston, SC USA; 3 H. Lee Moffitt Cancer Center & Research Institute, Tampa, FL USA; 4 Stanford University School of Medicine, Stanford, CA USA; 5 Peter O’Donnell Jr. School of Public Health, UT Southwestern Medical Center, Dallas, TX USA; 6 Cleveland Clinic Taussig Cancer Center, Cleveland, OH USA; 7 University of Maryland Marlene and Stewart Greenebaum Comprehensive Cancer Center, Baltimore, MD USA; 8 Levine Cancer Institute, Charlotte, NC USA; 9 The University of Kansas Medical Center, Kansas City, KS USA; 10 Virginia Commonwealth University Massey Cancer Center, Richmond, VA USA; 11 The University of Utah Huntsman Cancer Institute, Salt Lake City, UT USA; 12 The University of Texas MD Anderson Cancer Center, Houston, TX USA; 13 City of Hope National Medical Center, Duarte, CA USA; 14 Dana-Farber Cancer Institute, Boston, MA USA; 15 Mayo Clinic, Rochester, MN USA

**Keywords:** Myeloma, Risk factors

Idecabtagene vicleucel (ide-cel) the first FDA-approved gene therapy for relapsed refractory multiple myeloma (RRMM). However, its administration presents challenges in logistical management, selecting bridging therapy (BT), and customizing T-cell manufacturing, a complex process spanning several weeks [1]. In the KarMMa trial, BT was allowed but limited to specific prior drug classes (e.g., dexamethasone, cyclophosphamide (Cy), daratumumab, carfilzomib, bortezomib, or pomalidomide) [2]. This study delves into the impact of different BT on outcomes for RRMM patients undergoing standard of care (SOC) ide-cel treatment, aiming to clarify their role in CAR T therapy outcomes.

This retrospective multicenter study observed RRMM patients receiving ide-cel treatment at 11 U.S. medical centers within the U.S. Myeloma Immunotherapy Consortium. All RRMM patients treated with ide-cel from 5/2021 to 5/2022, were included. BT was defined as systemic treatment between leukapheresis and CAR-T infusion, categorized into: Selinexor (Selinexor-containing regimens); alkylator (alkylator-based); PI combos (sole or combined proteasome inhibitor (PI) therapy); IMiD +/- mAb combos (steroids with/without immunomodulatory (IMiDs) and/or Monoclonal Antibodies (mAb).

High-risk cytogenetics included del (17p), t(4;14), and t(14;16) pre-CAR-T. Cytokine Release Syndrome (CRS) and Immune Effector Cell-Associated Neurotoxicity Syndrome (ICANS) were graded by American Society for Transplantation and Cellular Therapy criteria [3]. Hematologic toxicity was assessed using Common Terminology Criteria for Adverse Events version 5.0 [4]. Disease response followed the revised International Myeloma Working Group criteria [5].

Subgroup comparisons employed chi-square/Fisher’s exact tests for categorical, *t*-tests/ANOVA for continuous variables. Survival analysis used Kaplan-Meier/Cox proportional hazards model. Stepwise Cox regression identified significant variables associated with overall survival (OS) and progression free survival (PFS), based on *p*-values < 0.2 from initial analysis. SAS (v9.4) and IBM SPSS (v29.0) conducted all statistical analyses.

Of 214 ide-cel patients, 170 (79%) underwent BT, encompassing 12% Selinexor, 45% alkylator, 15% PI combos, 18% IMiD +/- mAb combos, and 11% other therapies (e.g., belantamab mafodotin, focal radiation). Forty-four patients (21%) did not receive BT (no-BT). The median BT duration was 1 month (range, 1-7). While most had 1–2 months, one patient had 7 months with 4 treatments. BT patients showed poorer eastern Cooperative Oncology Group Performance Status (ECOG), higher Revised International Staging System (R-ISS) (2-3), elevated ferritin ( > 300 ng/mL), and C-reactive protein (CRP) ( > 5 mg/L) at lymphodepleting chemotherapy (Supplementary 1). IMiD +/- mAb combos had less extramedullary disease (EMD) (*p* = 0.052), and alkylator groups often received alkylator as last pre-apheresis treatment (*p* < 0.001). No differences in ECOG, R-ISS, ferritin, CRP, or high-risk cytogenetics among BT subgroups emerged. Median prior therapy count, and penta-refractory status were similar between BT and no-BT groups, including BT subgroups.

No significant differences in incidence/severity of CRS between BT and no-BT groups were noted, consistent across BT subgroups. Although ICANS incidence was higher in the BT, it lacked statistically significance (any grade: 21% vs. 14%, *p* = 0.070; grade ≥2: 13% vs. 2%, *p* = 0.070). Within the BT subgroup, Selinexor showed notably higher ICANS (grade ≥2) at 38% compared to others (alkylator 9%, PI combos 0%, IMiD +/- mAb combos 17%; *p* = 0.023). Notably, patients receiving Selinexor had no known central nervous system pathology. The reason is unclear, but endothelial dysfunction and increased blood-brain barrier permeability [6] might contribute to the higher ICANS rate in this subgroup.

BT patients had longer hospital stays than no-BT (median: 10 vs. 8 days; *p* < 0.001). Alkylator and Selinexor had the lengthiest stays (median: 11 and 10.5 days, respectively), followed by PI combos (9 days) and IMiD±mAb combos (9 days).

At day 7 post-infusion, no cytopenia differences emerged between BT and no-BT groups. However, Selinexor caused higher-grade anemia (*p* < 0.001) and thrombocytopenia (*p* = 0.043).

At 3 months post-infusion, BT showed higher rates of any-grade neutropenia (47% vs. 27.5%, *p* = 0.030), and anemia (79% vs. 50%, *p* < 0.001), with no significant differences in severe cases (≥3). Thrombocytopenia didn’t differ between groups or subgroups (supplementary 2).

At 3 months post-CAR-T, response didn’t differ between BT and no-BT (*p* = 0.802 for overall response rate (≥ partial response (PR); *p* = 0.208 for complete response (CR), consistent across BT subgroups (supplementary 3).

Median follow-up: 9.7 months (range: 0.2–19.5). Median PFS: 8.16 months (95% confidence interval (CI): 6.61–9.31), OS: not reached (NR). 1-year PFS and OS rates: 36 and 63%, respectively. BT patients showed inferior PFS (6.68 vs. 11.48 months in no-BT, *p* = 0.007) and OS (13.85 vs. NR months in no-BT, *p* = 0.002) (Table 1, Fig. 1A, B).

**Table 1:** Outcome based on Bridging therapy.

	No.	Bridging therapy (BT)			No.	Bridging therapy type					
Outcome		Yes (*n*, %)	No (*n*, %)	*P*-value		No BT (*N* = 44)	Selinexor (*N* = 21)	Alkylator (*N* = 76)	PI combos (*N* = 25)	IMiD Combo (*N* = 30)	*P*-value
**Best CR or better at 3 months**	172	54 (41%)	21 (52.5%)	0.208	157	21 (52.5%)	5 (26%)	22 (42%)	7 (37%)	12 (44%)	0.412
**Best ORR at 3 months**	172	111 (84%)	35 (87.5%)	0.802	157	35 (87.5%)	15 (79%)	45 (87%)	15 (79%)	26 (96%)	0.387
**MRD negativity at 3 months**	95	52 (72%)	20 (87%)	0.175	119	20 (89%)	8 (61.5%)	13 (56.5%)	5 (83%)	18 (90%)	0.042
**Best CR or better at 6 months**	126	44 (47%)	17 (53%)	0.547	119	17 (53%)	4 (33%)	18 (43%)	6 (54.5%)	12 (54.5%)	0.666
**Best ORR at 6 months**	126	76 (81%)	30 (94%)	0.099	85	30 (94%)	10 (83%)	31 (74%)	10 (91%)	19 (86%)	0.199
**PFS**	213			**0.007**							**0.010**
Median PFS (95% CI), months		6.68 (5.79–8.49)	11.48 (9.05–17.73)			11.48 (9.05–17.73)	9.77 (4.11–13.88)	6.51 (4.18–8.16)	6.41 (2.70–12.50)	12.01 (5.79-NR)	
**OS**	213			**0.002**							**0.001**
Median OS (95% CI), months		13.85 (11.97- NR)	NR			NR-NR	NR-NR	11.97 (8.91–15.53)	NR-NR	NR-NR	

Best response: ≥ complete response, best overall response rate ≥ partial response, *CI* Confidence interval, *NR* not reached. *PFS* progression-free survival, *OS* Overall survival, *BT* bridging therapy, Selinexor containing Selinexor as part of regimen, Alkylator containing alkylator as part of regimen, *PI* combos containing proteasome inhibitor alone or in combination, *IMiD* combos containing steroid ± immunomodulator (IMiDs) ± monoclonal antibodies (MoA), no BT no bridging therapy, *P*-values ≤ 0.05 are shown in bold.

**Fig. 1:**
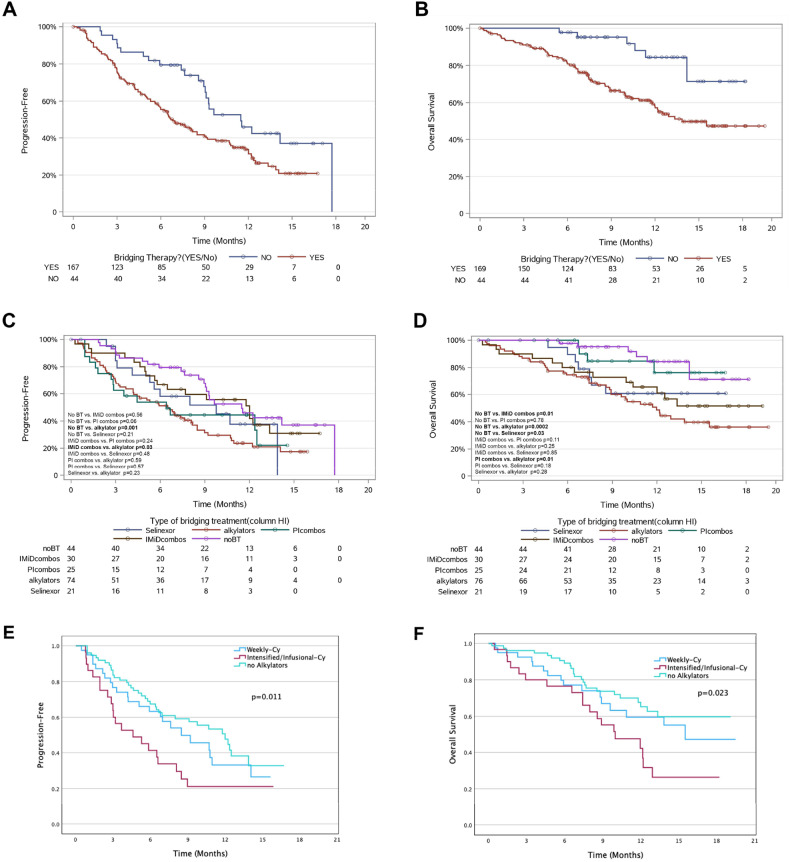
Survival based on bridging therapy strategy. Kaplan-Meier plots estimates of PFS **A** and OS **B** in RRMM patients treated with idecabtagene vicleucel, between those with and without bridging therapy. Kaplan-Meier estimates of PFS **C** and OS **D** categorized by bridging therapy type (no-BT, Selinexor, alkylator, PI combos, IMiD±mAb combos). Kaplan-Meier estimates of PFS **E** and OS **F** categorized based on cyclophosphamide dosing and exposure as bridging therapy.

IMiD±mAb combos showed comparable PFS to no-BT (median PFS: 12.01 months (95% CI, 5.79-NR) vs. 11.48 months (95% CI, 9.05–17.73); *p* = 0.56). Other therapies exhibited varying PFS durations in comparison with IMiD±mAb combos: Selinexor (9.77 months, 95% CI, 4.11–13.88; *p* = 0.48), PI combos (6.41 months, 95% CI, 2.70–12.50; *p* = 0.24), and alkylator (6.51 months, 95% CI, 4.18–8.16; *p* = 0.030) (Fig. 1C).

Alkylator use resulted in inferior OS (median OS: 11.97 months, 95% CI: 8.91–15.53) compared to others (NR) (*p* = 0.001) (Fig. 1D). Seventy deaths occurred: 49 disease-related, 4 due to CRS/ICANS, 1 CAR-T myocarditis, 10 infection-related, rest unrelated.

The univariate Cox regression for OS and PFS confirmed inferior outcomes among patients with ECOG 2–4, R-ISS 3, EMD, last pre- apheresis treatment with BCMA-targeted therapy, alkylator-based BT, and lack of CR by 3 months. Stepwise Cox regression revealed a significant association of BT with worse PFS (*p* = 0.027), particularly with alkylator use ((hazard ratio (HR) = 2.54, 95% CI (1.39–4.62); *p* = 0.002). A similar trend, though not statistically significant, was noted in Selinexor and PI combos. Response less than CR [(PR/VGPR (very good PR): HR = 2.05, *p* = 0.019; stable/progression disease (SD/PD): HR = 31.32; *p* < 0.0001)] was associated with inferior PFS. For OS, responses less than CR (HR = 3.91; *p* = 0.0002) and high-risk cytogenetics (HR = 5.77; *p* = 0.008) were associated with poorer outcomes.

Our analysis highlighted alkylators as linked to worse PFS despite no initial differences in tumor burden or inflammatory markers among BT subgroups. Therefore, we examined alkylator types to gauge their impact. In alkylator BT, 92% received Cy, others had oral melphalan or melphalan flufenamide; bendamustine was not used. Among Cy-treated patients, 43% had intensified/infusional (hyperfractionated Cy-based (hyperCy), DCEP, PACE), and 57% had weekly doses (e.g., CyBorD, KCD, etc.). Intensified/infusional vs. weekly Cy showed no significant difference in median PFS (4.61 vs. 8.49 months, *p* = 0.089) or OS (10 vs. 15.5 months, *p* = 0.11). Comparison of weekly Cy with no-alkylator group showed no significant differences in median PFS (*p* = 0.3) or OS (*p* = 0.3).

However, intensified/infusional resulted in poorer outcomes compared to no-alkylator group (median PFS: 4.6 vs. 12.0 months, *p* = 0.002; median OS: 10 vs. NR months, *p* = 0.006) (Fig. 1E, F, supplementary 4). In a recent analysis [7] of CAR T-cell therapies for RRMM, grouping BTs by Cy usage (solely hyperCy-based, weekly Cy, no Cy/alkylator) demonstrated comparable CRS, ICANS rates, and PFS. Notably, hyperCy group patients experienced prolonged platelet recovery and lower OS, possibly due to disease aggressiveness.

Note that, in retrospective studies, assessing tumor burden and baseline markers offers only a snapshot, potentially missing full disease progression or patient status. Earlier studies suggest chemotherapy’s lasting impact on T cells, potentially affecting their function [8, 9]. While specific pre-apheresis T cell quality data is lacking and no observed PFS difference based on pre-apheresis alkylator exposure, pre-collection T cell quality might affect outcomes. Moreover, alkylator use in aggressive disease contexts may hinder sustained treatment response, affecting outcomes.

Among 170 BT patients, 12% responded to BT (*n* = 21; 1 CR, 8 VGPR, 12 PR); response rates didn’t significantly differ among subgroups (*p* = 0.503). BT response showed no significant association with CRS, ICANS rates, or 3-month post CAR-T response (*p* = 1.00, *p* = 1.00, *p* = 0.425, respectively). Median PFS didn’t significantly differ based on BT response (6.51 months in ≥PR vs. 8.48 months in SD/PD; *p* = 0.6). Despite an 88% BT rate in the KarMMa trial, the response remained low at 5% (*n* = 5) [2]. This implies that BT may not confer superiority or necessity for stable patients. Further evaluation through prospective studies or extensive registry analysis with larger cohorts is essential.

In our study, only 4 patients received BCMA-directed antibody-drug conjugate as BT. They were excluded from subgroup analysis due to limited cases and significantly inferior PFS. No patients received bispecific T-cell engagers as BT.

Limited by retrospective design and lacking BCMA data, reduced PFS might relate to decreased tumor BCMA expression [10]. Our previous data showed lower PFS post BCMA-targeted therapy [11], and hinted at compromised ide-cel efficacy within six months of such treatment [12].

The retrospective nature of our study introduces potential selection biases. Additionally, smaller sample sizes within certain treatment regimens limit drawing definitive conclusions, alongside the probable influence of disease aggressiveness on BT choice.

In summary, patients with no-BT before ide-cel showed prolonged PFS and OS, possibly indicating less aggressive disease. Conversely, alkylator-based BT resulted in inferior PFS and OS compared to other BT types or no-BT approaches. This trend might relate more to refractory myeloma than directly to alkylators, proposing the potential benefit of early CAR T-cell therapy before standard treatment resistance. Tailoring BT based on patient history, toxicity risks, and disease traits is crucial. While reconsidering the necessity of BT in stable disease, considering the risk of toxicity due to inadequate cytoreduction remains crucial. However, caution with intensified/infusional Cy, if feasible, is advisable. Personalized assessments are pivotal in selecting bridging therapy.

### Supplementary information

Supplementary 1

Supplementary 2

Supplementary 3-4

## Data Availability

The data that support the findings of this study are available from the corresponding author upon reasonable request.
