# Supplementary material for: Real-world impact of bridging therapy on outcomes of ide-cel for myeloma in the U.S. Myeloma Immunotherapy Consortium
Source: Blood Cancer J. 2024 Apr 12;14(1):63. doi: 10.1038/s41408-024-00993-0 (PMC11015040; doi:10.1038/s41408-024-00993-0)
Supplement: Supplementary file 3 — Supplementary 3-4 [file 41408_2024_993_MOESM3_ESM.pdf]

Supplementary 3: Response. Efficacy with idecabtagene vicleucel in patients with relapsed/refractory multiple myeloma according to bridging therapy.

Abbreviation: CR: complete response; sCR: stringent CR; VGPR: very good partial response; PR: partial response; Selinexor: containing Selinexor as part of regimen; Alkylator: containing alkylator as part of regimen; PI combos: containing proteasome inhibitor alone or in combination; IMiD +/- mAb combos: containing steroid ± immunomodulator (IMiDs) ± monoclonal antibodies (MoA); no-BT: no bridging therapy.

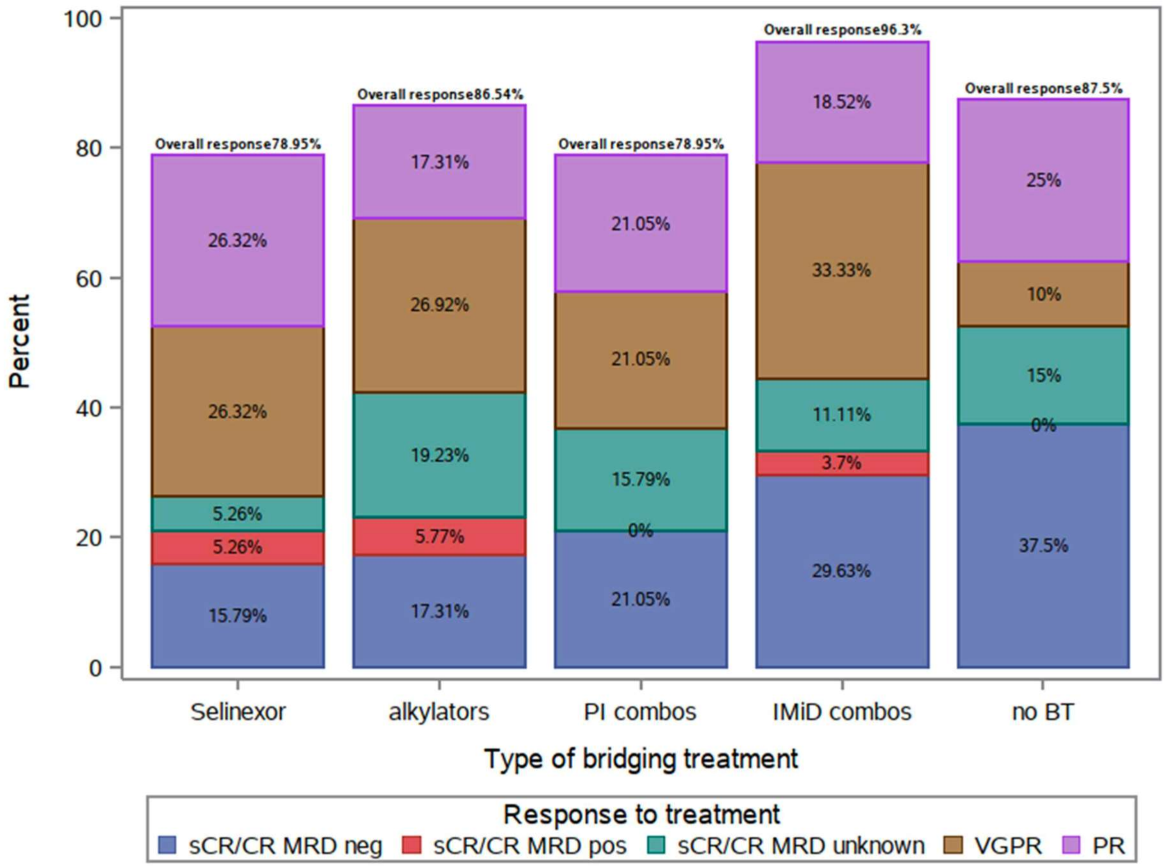

Supplementary 4. Survival outcome based on cyclophosphamide dosing and exposure as Bridging therapy.

Outcome	No-alkylator (N=78)	Weekly Cy (N=40)	Intensified/Infusional Cy (N=30)	P-value
**PFS**				**0.011**
Median PFS (95% CI), months	12.0 (8.9-15.0)	8.4 (4.4-12.4)	4.6 (1.2-7.9)	
**OS**				**0.023**
Median OS (95% CI), months	NR-NR	15.5 (NR-NR)	10.0 (5.6-14.3)	

Abbreviations: Cy: Cyclophosphamide; CI: Confidence interval; NR: not reached. PFS: progression- free survival; OS: Overall survival; weekly Cy: regimen such as (CyBorD, KCD, etc.); Intensified/ Infusional Cy (DCEP (n=14), PACE-based (n=9), Hyperfractionated Cy-based) (n=7); no-alkylator: any regimen without alkylator or BCMA-targeted therapy; P-values ≤ 0.05 are shown in bold.
